# Microinvasive Cervical Cancer as a Model for Therapeutic De-Escalation in Gynecologic Oncology

**DOI:** 10.3390/diagnostics16213501

**Published:** 2026-10-26

**Authors:** Maria Teresa Bruno, Carla Lo Giudice, Alessia Pagana, Teresa Calareso, Sara Boemi, Alessandra Fiorito, Fabio Bonaffini

**Affiliations:** 1 Department of General Surgery and Medical Surgery Specialties, Gynecological Clinic Policlinico Rodolico, University of Catania, 95123 Catania, Italy; carla.logiudice@studium.unict.it (C.L.G.); alessia.pagana@studium.unict.it (A.P.); teresa.calareso@studium.unict.it (T.C.); 2 Servizio di Radiologia, 97100 Ragusa, Italy; saraboemi@unimi.it; 3 UO di Ginecologia e Ostetricia, Ospedale “SS. Addolorata”, 95033 Catania, Italy; alessandra.fiorito@aspcatania.it (A.F.); fabio.bonaffini@aspcatania.it (F.B.)

**Keywords:** microinvasive cervical cancer, FIGO IA1, FIGO IA2, therapeutic de-escalation, conization, fertility-sparing treatment, lymphovascular space invasion, sentinel lymph node, HPV, cervical adenocarcinoma

## Abstract

Background: Microinvasive cervical cancer represents the earliest stage of stromal invasion and is associated with excellent oncological outcomes. Its clinical relevance arises from the potential disproportion between the limited extent of invasive disease and the morbidity of radical treatment, particularly in young women wishing to preserve fertility. Increasing evidence supports a transition from stage-based surgical radicality toward individualized, risk-adapted management. This narrative review critically examines microinvasive cervical cancer as a distinct clinical model of therapeutic de-escalation, with particular attention to the boundaries between evidence-supported conservative treatment and areas in which uncertainty persists. Methods: A narrative review of PubMed/MEDLINE was conducted for studies published between January 2010 and March 2026. Evidence concerning FIGO stages IA1 and IA2 was analyzed, focusing on diagnostic conization, pathological risk factors, lymphovascular space invasion (LVSI), margin status, histological subtype, fertility-sparing treatment, sentinel lymph node (SLN) assessment, ultrastaging, and follow-up. Studies including broader early-stage cervical cancer populations were considered only when stage IA results were separately available or clinically transferable to microinvasive disease, and were interpreted as indirect evidence when appropriate. Results: Accurate conization represents the cornerstone of the diagnosis and initial risk stratification of microinvasive cervical cancer, providing essential information on depth of stromal invasion, margin status, LVSI, histological subtype and multifocality. However, optimal risk assessment should integrate pathological findings with clinical staging, appropriate imaging and, when indicated, nodal assessment. FIGO stage alone does not adequately define individual risk, while imaging itself cannot reliably exclude occult small-volume nodal disease; therefore, the relative contribution of pathology, imaging and surgical nodal staging should be tailored to the individual risk profile. In FIGO IA1 disease without LVSI and with negative margins, conization alone provides excellent oncological control and lymph node staging is generally unnecessary. LVSI should be considered a risk modifier that may influence nodal assessment rather than an automatic indication for more radical cervical surgery. In IA1 with LVSI and IA2 disease, SLN biopsy may provide nodal staging with lower morbidity than systematic lymphadenectomy, although evidence specifically generated in microinvasive tumors remains limited. Fertility-sparing treatment is oncologically appropriate in carefully selected patients, whereas simple hysterectomy represents an adequate definitive option after completion of reproductive plans. Microinvasive adenocarcinoma requires specific consideration because of its endocervical location, potential multifocality, and the biological distinction between HPV-associated and HPV-independent histotypes. Major uncertainties remain regarding the prognostic significance of LVSI and low-volume nodal disease, the optimal indications for SLN assessment, and the limits of exclusive conservative treatment in IA2 disease. Conclusions: Microinvasive cervical cancer should not be regarded simply as the earliest anatomical stage of cervical cancer, but as a distinct clinical setting in which diagnostic precision may enable oncologically safe therapeutic de-escalation. The available evidence supports a shift from maximum surgical radicality toward the minimum effective treatment, provided that accurate histopathological assessment, appropriate patient selection, and specialized follow-up are ensured. Importantly, several recommendations for stage IA—particularly for IA2 disease and nodal management—still rely partly on evidence extrapolated from broader early-stage cervical cancer populations. Prospective multicenter studies and dedicated stage IA registries are therefore needed to define more precisely the boundaries within which therapeutic de-escalation can be safely applied.

## 1. Introduction

Cervical cancer remains a major global health burden. According to the latest GLOBOCAN 2024 estimates, approximately 604,000 new cases and 280,000 deaths occurred worldwide in 2024, with the highest incidence and mortality rates observed in low- and middle-income countries [1,2]. The distribution of the disease remains highly unequal and reflects differences in access to HPV vaccination, screening, and treatment of precancerous lesions. In countries with organized screening programs, the use of HPV testing has increased the identification of high-grade lesions and early-stage carcinomas [1,2,3].

Microinvasive cervical cancer represents a transitional stage between high-grade squamous intraepithelial neoplasia (HSIL/CIN3) and overt invasive carcinoma. It is defined exclusively histologically and includes International Federation of Gynecology and Obstetrics (FIGO) stages IA1 and IA2, characterized by stromal invasion of ≤3 mm and >3 to ≤5 mm, respectively [4]. The oncological risk is generally low, but not uniform: lymphovascular space invasion (LVSI), margins, histological type, multifocality and lymph node status modify the prognosis and must integrate the anatomical staging [5,6].

Management has therefore shifted from systematic radicality to risk stratification. Conization, simple hysterectomy, and staging by sentinel lymph node biopsy now allow for limiting morbidity, preserving fertility when desired, and maintaining adequate oncological control in selected patients [6,7]. This narrative review summarizes the evidence on diagnosis, pathology, treatment, fertility-sparing, LVSI, sentinel lymph node biopsy, and follow-up, distinguishing data specific to stage IA from those extrapolated from early stages with larger tumor volume.

The clinical interest in this tumor stems precisely from the potential disproportion between the small size of the invasive focus and the impact of radical treatment. This issue is particularly relevant in younger women, in whom earlier detection of cervical neoplasia—potentially reflecting both changes in disease epidemiology and advances in screening and diagnostic strategies—may result in a longer lifetime burden of treatment-related morbidity. Given the excellent expected survival, not only oncological cure but also fertility, bladder and sexual function, body image, and quality of life become central considerations. De-escalation, however, does not equate to indiscriminate simplification: it presupposes a reliable histological diagnosis, the exclusion of risk factors, and the possibility of ensuring specialist monitoring. Against this background, this review critically examines microinvasive cervical cancer as a distinct clinical model of therapeutic de-escalation, distinguishing evidence generated specifically in FIGO stage IA from that extrapolated from broader early-stage cervical cancer populations and identifying both the evidence-supported boundaries and the unresolved areas of conservative management.

## 2. Materials and Methods

This narrative review was based on a targeted literature search conducted in PubMed/MEDLINE to identify clinically relevant evidence on the diagnosis and management of microinvasive cervical cancer. The search focused primarily on publications from January 2010 to October 2026, while earlier landmark studies were retained when considered relevant to the historical development of staging or treatment strategies. Search terms included combinations of “microinvasive cervical cancer”, “FIGO IA1”, “FIGO IA2”, “cervical conization”, “fertility-sparing treatment”, “lymphovascular space invasion”, “sentinel lymph node”, “ultrastaging”, “micro-metastases”, “isolated tumor cells”, “adenocarcinoma”, and “therapeutic de-escalation”. Additional recent terms related to emerging diagnostic approaches, including “liquid biopsy”, “circulating tumor DNA”, “circulating HPV DNA”, “microRNA”, “miR-21”, “single-cell RNA sequencing”, and “spatial transcriptomics”, were considered during revision.

Priority was given to international guidelines, systematic reviews and meta-analyses, prospective studies, and large retrospective cohorts. Studies specifically addressing FIGO stage IA disease were preferentially considered; when stage IA-specific evidence was limited, data from broader early-stage cervical cancer populations were included and their indirect applicability to microinvasive disease was explicitly acknowledged. Reference lists of relevant articles and guidelines were also screened to identify additional pertinent publications.

Because the objective was to provide a critical narrative synthesis rather than a systematic review or meta-analysis, no formal study-quality scoring, risk-of-bias assessment, or quantitative evidence synthesis was performed. The selected evidence was critically interpreted according to its relevance to diagnostic accuracy, oncological safety, fertility preservation, treatment-related morbidity, and therapeutic de-escalation.

This methodological approach introduces the central theme of the review: the quality of the therapeutic decision depends first and foremost on the accuracy with which microinvasion is defined.

## 3. Definition, Diagnosis and Anatomopathological Parameters

### 3.1. Evolution of the Classification

The 1994 FIGO classification, maintained in 2009, defined IA1 as stromal invasion ≤ 3 mm and IA2 as invasion > 3 and ≤5 mm, setting a horizontal cutoff of 7 mm for both [8]. The 2018 FIGO revision eliminated horizontal extent because it was poorly reproducible and of limited independent prognostic value, maintaining depth as a discriminating criterion [4]. The removal of the 7 mm horizontal extent criterion from the 2018 FIGO definition of stage IA was based on several pathological considerations. Horizontal tumor extent showed limited reproducibility, particularly in fragmented or multifocal specimens, and the previous 7 mm threshold was considered relatively arbitrary, with insufficient evidence supporting an independent association with lymph-node involvement or prognosis [9,10]. Importantly, removal of horizontal extent from staging does not imply that this parameter has become pathologically irrelevant; horizontal extent, multifocality, and the number of involved tissue blocks should still be documented when assessable [9,11].

The implications also differ according to histological type. In squamous cell carcinoma, depth of stromal invasion remains the principal measurement for assigning stage IA1 or IA2, although superficially spreading tumors illustrate a potential limitation of disregarding horizontal extent entirely [9]. In endocervical adenocarcinoma, assessment may be more challenging because of glandular architecture, multifocal growth, and difficulties in reconstructing tumor extent across multiple tissue fragments. In this setting, summation of horizontal measurements from separate specimens may artificially overestimate tumor size [11]. Nevertheless, available evidence indicates that horizontal tumor extent has limited independent prognostic significance in stage I endocervical adenocarcinoma, supporting its removal as a formal FIGO staging criterion [10].

The 2020 WHO classification also distinguishes HPV-associated carcinomas from HPV-independent ones, a distinction that is particularly relevant in adenocarcinoma, since HPV-independent histological types have more aggressive behavior and are not candidates for conservative treatment [5,8]. Morphological definition must therefore precede any therapeutic choice.

The evolution of the classification reflects a broader conceptual shift. Historical definitions tended to describe microinvasion through multiple dimensional parameters; the current approach recognizes that depth, while essential for stage assignment, does not alone capture biological risk. Two tumors with identical stromal invasion may differ in LVSI, histology, margins, and growth pattern. The FIGO staging must therefore be interpreted in conjunction with the complete pathology report, preventing the stage number from becoming the sole therapeutic criterion.

### 3.2. From HPV Testing to Diagnostic Conization

Microinvasive carcinoma is rarely clinically recognizable. In most cases, it emerges during further investigation of a positive HR-HPV test associated with HSIL, ASC-H or AGC. The HPV test has greater sensitivity than cytology for high-grade lesions, but no screening test can establish the depth of invasion [3]. Colposcopy represents a fundamental step in the diagnostic process, allowing the lesion to be located and guiding the biopsy sample. However, it is important to underline that microinvasive carcinoma does not have pathognomonic colposcopic characteristics: the signs described by Bornstein [12] may suggest a severe lesion, but are not pathognomonic of microinvasion. Targeted biopsy represents the first histological assessment, but has intrinsic limitations in the diagnosis of microinvasive carcinoma. The reduced extent of stromal infiltration makes it possible to incompletely sample the lesion, resulting in an underestimation of the real depth of invasion.

Several studies have shown that a significant proportion of microinvasive carcinomas are diagnosed only on the conization specimen after a biopsy initially interpreted as HSIL/CIN3. Therefore, biopsy should be considered a preliminary diagnostic tool, while conization represents the reference examination for definitive staging [5,6].

Conization therefore remains the reference test because it allows the evaluation of the entire epithelium-stroma interface, measurement of invasion, documentation of LVSI and multifocality and definition of margins. The sample must be intact, oriented and entirely included; the depth is measured from the basement membrane of the epithelium of origin to the deepest point of invasion. In cases with multiple foci, the deepest foci are considered, without adding the areas [4,5,6]. This measurement method is now universally accepted by current international anatomical-pathological classifications and recommendations and represents the essential prerequisite for correct staging [4,5,6,13].

The quality of the excisional procedure is crucial. Cone fragmentation, thermal artifacts, and inadequate orientation can make measurement uncertain or prevent margin assessment. The technique should be chosen based on the location and extent of the lesion, the visibility of the squamocolumnar junction, and the suspected glandular involvement. LEEP can provide an adequate specimen when performed in a single piece and with limited thermal damage; cold-knife conization remains particularly useful in endocervical lesions, adenocarcinoma in situ, and when a particularly rigorous morphometric evaluation is required [6,14,15,16,17,18,19,20,21]. The report should specify histotype and grade, depth of invasion, any horizontal extension, number of foci, presence or absence of LVSI, and distance from the endocervical, ectocervical, and deep margins. In equivocal cases, review by an experienced gynecologic pathologist reduces the risk of over- or understaging. This step is particularly important in distinguishing true stromal invasion from glandular involvement by HSIL, stromal reactions, and sampling artifacts [22].

### 3.3. Margins, LVSI, Histotype and Imaging

Negative margins for invasive carcinoma and HSIL are essential for considering therapeutic conization. Positive or non-evaluable margins require, when technically possible, a new excision to exclude more extensive disease. Hartman et al. [14], in 562 IA1 patients, showed that the height and quality of the cone influence the completeness of the margins and the reliability of the staging; the objective, however, is not to use a standardized cone for all patients, but to perform the minimum excision capable of obtaining an adequate diagnosis and radicality.

An additional emerging consideration is the biological information provided by the tumor microenvironment beyond conventional histopathological parameters. In cervical cancer, microRNA-21 (miR-21) has been shown to be preferentially expressed in the tumor-associated stromal compartment, supporting the biological relevance of tumor–stroma interactions [23]. More recent single-cell RNA sequencing and spatial transcriptomic studies have further demonstrated marked spatial heterogeneity and complex interactions between malignant epithelial cells, stromal cells, and immune populations in cervical cancer [24,25]. However, these molecular findings have not yet been validated as criteria for determining the extent of cervical excision in FIGO stage IA disease. In particular, there is currently no clinical evidence that extending conization beyond histologically adequate margins to remove additional peritumoral stroma improves oncological outcomes. Future studies integrating spatial molecular profiling with conventional pathological parameters may determine whether stromal biomarkers can refine risk stratification and guide the extent of conservative surgery.

LVSI increases the probability of lymph node involvement, but does not modify the FIGO stage and should not be interpreted in isolation. Histological type, depth, margins and clinical features also contribute to the decision [6,26,27]. Imaging has a limited role in microinvasion: MRI, CT and PET/CT are not routinely indicated in correctly defined IA1. MRI or expert ultrasound become useful when there is doubt of residual disease, endocervical lesion, candidacy for trachelectomy or suspicion of a higher stage [6,28,29]. Histopathology and imaging should therefore be considered complementary rather than competing components of risk assessment. Histopathological examination of an adequate conization specimen remains essential for confirming microinvasion and defining the depth of stromal invasion, margin status, LVSI, histological subtype and multifocality [30]. Imaging, in contrast, provides information beyond the excised specimen and may help identify residual cervical disease, local extension or findings suggesting a higher stage when clinically indicated [30,31]. However, the absence of suspicious findings on imaging does not reliably exclude occult microscopic or low-volume nodal disease [32]. Therefore, negative imaging should not replace nodal assessment when this is indicated by the pathological risk profile [30,32]. An integrated approach combining histopathology, clinical assessment, selective imaging and, when appropriate, surgical nodal staging provides the most reliable basis for individualized treatment and safe therapeutic de-escalation [30].

Beyond conventional imaging and routine histopathological assessment, emerging molecular pathology approaches may provide complementary information on the biological transition from intraepithelial to invasive cervical disease. Immunohistochemical and in situ hybridization studies have demonstrated changes in biomarkers including p16, Ki-67, PD-L1, and HPV E6/E7 RNA across cervical intraepithelial, microinvasive, and invasive lesions. FISH-based genomic signatures have also been investigated as predictors of lymph-node metastasis in early cervical cancer, although available evidence derives predominantly from tumors beyond FIGO stage IA and cannot currently be extrapolated to microinvasive disease. More recently, single-cell and spatial transcriptomic approaches have identified distinct epithelial programs associated with cervical cancer progression and, in spatial analyses, invasive-edge and tumor–stroma-interface programs involving epithelial–mesenchymal transition and matrix adhesion. These technologies are promising for refining biological risk stratification, but none has yet been validated to predict occult metastatic disease or to guide treatment decisions specifically in FIGO stage IA cervical cancer. Histopathological assessment of an adequate excisional specimen therefore remains the diagnostic reference standard.

The diagnosis of LVSI also requires caution, as retraction spaces and artifacts can mimic tumor emboli. Evaluation should be based on the presence of tumor cells in endothelial-lined spaces, ideally outside the primary invasion front; immunohistochemistry can be helpful in selected cases but does not replace morphological interpretation. A questionable finding should not, by itself, lead to increased surgical radicality, especially when fertility preservation or the indication for nodal staging depends on it. The main diagnostic and anatomopathological parameters relevant to the definition and risk stratification of microinvasive cervical cancer are summarized in Table 1.

Once the stage and risk profile have been confirmed, the process moves from diagnosis to the crucial therapeutic question: how much treatment is really necessary for that patient?

## 4. Treatment Guided by Risk and Reproductive Desire

### 4.1. Common Principles and Margin Assessment

Margin status is a central determinant of both diagnostic reliability and conservative management. Negative margins for invasive carcinoma and associated HSIL or AIS are required before conization can be considered definitive treatment. Positive or non-assessable margins compromise the reliability of pathological staging because a more extensive invasive component cannot be excluded. When technically feasible, repeat conization should therefore be considered to obtain an intact specimen with evaluable margins and to exclude residual or more extensive disease. The decision should take into account the site of margin involvement (endocervical, ectocervical, or deep), the presence of invasive carcinoma versus HSIL/AIS at the margin, residual cervical anatomy, histological type, LVSI, and reproductive goals. Margin status should not be interpreted in isolation, but together with depth of invasion, histotype, multifocality, and nodal-risk factors [6,11,12,33].

### 4.2. Stage-Specific Management: IA1 and IA2

Stage IA1

In IA1 without LVSI, with negative margins and favorable histological type, conization can be definitive in women wishing to have children; in women who have completed the reproductive plan, conization with surveillance or simple hysterectomy is appropriate, depending on age, comorbidities, preferences and reliability of follow-up [6,7]. Lymph node staging is not indicated. In the presence of LVSI, cervical surgery can remain conservative, but lymph node evaluation should be discussed, preferably by sentinel lymph node biopsy in an expert center [6] (Table 1).

Observational evidence is consistent. Hartman et al. [34] documented, with follow-up up to 20 years, an excellent prognosis in IA1. Hartman et al. [14] subsequently highlighted the relationship between cone size, margins and residual disease. Chen et al. [15] confirmed favorable oncological and obstetric outcomes after LEEP in patients without LVSI and with complete excision. Azzi et al. [33] finally found a low frequency of invasive residual disease after an adequate excisional procedure. These studies shift the focus from the technique used to the quality of the sample and correct selection.

After conservative treatment, surveillance should be adapted to the pathological risk profile. Persistent high-risk HPV infection, particularly persistence of the same genotype, is associated with an increased risk of residual or recurrent disease and warrants careful colposcopic follow-up [35]. Immediate hysterectomy should not be based on HPV persistence alone, but should be considered in the context of margin status, histology, residual disease, reproductive plans, and the feasibility of reliable surveillance.

In patients no longer wishing to become pregnant, the choice between surveillance after complete conization and simple hysterectomy is not exclusively oncological. Age, comorbidities, associated uterine pathologies, quality of the squamocolumnar junction, and availability for follow-up must be discussed with the patient. In the absence of LVSI, both strategies may be appropriate; what is not justified is the routine use of radical hysterectomy or lymphadenectomy in accurately staged IA1 disease [6,7].

Stage IA2.

In IA2 the probability of lymph node involvement is higher than in IA1; therefore, lymph node evaluation is indicated, generally by sentinel lymph node. In selected women with reproductive desire, negative margins, negative lymph nodes and favorable histology, wide conization or simple trachelectomy may be considered [6]. In women with no desire for pregnancy, simple hysterectomy combined with lymph node staging represents a de-escalation strategy supported by prospective evidence in low-risk early cervical cancer [36,37].

The cohort of Qian et al. [38] showed that depth of invasion and LVSI, more than surgical radicality per se, drive prognosis in microcarcinoma. However, the specific evidence for IA2 remains lower than that for IA1; the decision should therefore be multidisciplinary and clearly document the selection criteria.

Stage IA2 is therefore the area in which the decision-making process must be most individualized. Traditional radical hysterectomy with lymphadenectomy has been progressively scaled down, because parametrial involvement is exceptional in small tumors with both negative margins and lymph nodes. The clinical question is no longer whether all patients should receive radical surgery, but which ones can be treated with cervical excision or simple hysterectomy without losing safety. In this evaluation, LVSI, histology, residual volume, imaging in doubtful cases and the reliability of the center in lymph node mapping are relevant [6,36,37]. Table 2 shows a Risk-based therapeutic summary.

### 4.3. Fertility Preservation

Fertility preservation should be considered separately from stage-based surgical de-escalation. It is not simply a reduction in treatment, but an oncologically selected strategy aimed at maintaining reproductive potential without compromising cancer control. Eligibility should integrate stage, histological type, LVSI, margin status, nodal risk, and the patient’s reproductive goals. Zhang et al. [39], in their meta-analysis, reported low recurrence rates after conization or trachelectomy, despite the typical heterogeneity of observational studies. Wang et al. [40] confirmed favorable oncological and reproductive outcomes in a multicenter cohort. Gil-Ibañez et al. [41] instead showed that tumor volume remains a determining factor: the risk increases beyond carefully selected small-volume disease.

Before proposing conservative treatment, the real desire for pregnancy, understanding of the risks and adherence to follow-up must be verified. The ideal candidate has a squamous cell histology or usual HPV-associated adenocarcinoma, clear margins, absence of macroscopic residual disease and, when indicated, negative lymph nodes. Conization represents the least destructive procedure in IA1 and can be considered in carefully selected IA2 with lymph node staging. Simple trachelectomy is indicated when a wider cervical excision is necessary; radical trachelectomy should be reserved for exceptional conditions in which a less extensive procedure does not guarantee adequate margins [6,39,40,41,42,43,44,45,46,47,48,49,50].

The technical goal is to obtain clear margins while preserving as much healthy stroma as possible. Kyrgiou et al. [51] demonstrated a dose-effect relationship between depth of excision and preterm birth. Counseling should therefore include recurrence, need for prolonged follow-up, cervical stenosis, secondary infertility, late abortion, premature rupture of membranes and preterm birth.

Reproductive outcomes should not be described solely by the number of pregnancies achieved. It is necessary to distinguish between the actual desire to conceive, the time elapsed before attempting to conceive, the possible use of medically assisted procreation, and obstetric outcomes. Cervical stenosis can hinder both conception and surveillance; the reduction of the stroma increases the risk of cervical incompetence and prematurity. Collaboration with reproductive specialists and obstetricians experienced in high-risk pregnancies allows planning the timing of conception and the management of the pregnancy after treatment [42,43,52].

In this context, shared decision-making takes on substantial importance. A young woman may place a different value on uterine conservation than on the risk, even if low, of recurrence and the need for repeated check-ups. The team must balance conization, trachelectomy, and definitive treatment, avoiding both denying an oncologically appropriate conservative strategy and downplaying its implications. Informed consent should document the criteria that make the patient eligible and the conditions that, during follow-up, may require surgical completion.

### 4.4. Simple Hysterectomy After Completion of the Reproductive Project

In low-risk IA1, simple hysterectomy is definitive and avoids parametriectomy, extended ureteral dissection and increased urinary, intestinal and sexual morbidity. It is not mandatory when complete conization has been achieved, but it is a reasonable option in women who do not wish to become pregnant, especially in the presence of difficult follow-up or residual cervical pathology [6,7].

The SHAPE trial by Plante et al. [36], although including low-risk tumors up to 2 cm and not only microinvasive ones, demonstrated the oncological non-inferiority of simple hysterectomy compared to radical hysterectomy, with lower urological morbidity. The GOG-0278 trial by Covens et al. [37] further supported the feasibility of non-radical surgery in stages IA1 with selected LVSI, IA2 and IB1. These results strengthen the de-escalation, but do not eliminate the need for adequate preoperative conization and correct lymph node evaluation when indicated.

The clinical significance of these studies must be interpreted from a microcarcinoma perspective. SHAPE provided the strongest evidence against systematic parametriectomy in low-risk cervical cancer, showing that reducing radicality resulted in measurable functional benefits. GOG-0278 extended the observation to non-radical surgery and fertility outcomes. Due to the fact that the populations did not entirely coincide with stage IA, these trials do not replace the microcarcinoma-specific evidence, but they make it increasingly difficult to justify radical surgery in a patient with microscopic disease, negative margins, and adequate nodal evaluation [36,37]. The potential benefits of therapeutic de-escalation may also extend beyond the reduction of surgical morbidity and preservation of quality of life. A cost-effectiveness analysis from the GCIG/CCTG CX.5/SHAPE trial showed that simple hysterectomy was less costly and slightly more effective in terms of quality-adjusted life-years than radical hysterectomy in women with low-risk early-stage cervical cancer. These findings suggest that, when oncologically appropriate, reducing surgical radicality may also decrease healthcare resource utilization. However, dedicated health-economic analyses specifically focused on FIGO stage IA disease are still lacking and should be incorporated into future prospective studies [53].

### 4.5. Microinvasive Adenocarcinoma

In adenocarcinoma, patient selection should be more cautious because of the endocervical location of the lesion, the potential for multifocality, and the distinction between HPV-associated and HPV-independent histotypes. Cervical glandular disease is also particularly associated with HPV18, a genotype that is significantly more prevalent in adenocarcinoma and adenocarcinoma in situ than in squamous lesions. This association is also particularly relevant from a diagnostic perspective, since HPV18 tends to be less frequently detected in precancerous lesions relative to its representation in invasive carcinomas, potentially contributing to the lower sensitivity of screening for glandular lesions.

The distribution of HPV genotypes, however, varies according to the population and the type of cervical lesion considered. In Italian series, HPV16 is the predominant genotype, whereas HPV31 emerges as one of the most frequent types, particularly in CIN2/3 lesions; in invasive carcinomas, by contrast, HPV18 again ranks prominently after HPV16 [54,55,56].

In US populations, HPV16 remains the most prevalent genotype, whereas HPV18 shows a particularly strong association with glandular disease: in the ATHENA trial, HPV18 accounted for approximately half of the cases of adenocarcinoma in situ and invasive carcinoma identified in the study [57].

These differences in HPV genotype distribution, also observed in our own experience, suggest that HPV genotyping may provide additional information for risk stratification and surveillance of glandular lesions, although, at present, genotype alone does not represent an independent criterion for selecting patients for fertility-sparing treatment.

Reynolds, Tierney, Keeney, Felix, Weaver, Roman, and Cliby [58] reported favorable outcomes in microinvasive adenocarcinoma managed according to stage and margin status, supporting the feasibility of conization in patients with FIGO stage IA1 HPV-associated adenocarcinoma, negative margins, and no lymphovascular space invasion (LVSI). Aggressive HPV-independent histotypes remain unsuitable for fertility-sparing treatment [5,6].

The concomitant presence of adenocarcinoma in situ makes evaluation of the endocervical margin essential and increases the importance of an intact specimen. So-called skip lesions and localization in the canal can reduce the reliability of superficial biopsies or fragmented excisions. In patients wishing to have children, a second conization may be preferable to an immediate hysterectomy when the first margin is not free and a new excision is technically safe. After completion of the reproductive plan, simple hysterectomy remains appropriate in favorable and correctly staged histotypes [5,6,58].

The choice of cervical extension concludes the local treatment; it remains to be determined, in high-risk cases, whether and how to explore the lymphatic system.

## 5. Lymph Node Staging

### Sentinel Lymph Node and Ultrastaging

In IA1 without LVSI, the lymph node risk is so low that it does not justify staging. In IA1 with LVSI and in IA2, sentinel lymph node mapping allows for prognostic information while reducing the morbidity of systematic lymphadenectomy [6]. In this setting, LVSI should be interpreted as a risk modifier rather than as an independent indication for more radical cervical surgery. Its principal clinical relevance is to refine the estimated risk of lymph-node involvement and, together with depth of invasion, histological subtype, margin status, and other pathological features, to guide the indication for sentinel lymph-node biopsy or other nodal assessment. Cao et al. [20], in 975 patients with stage IA-IIA disease, identified LVSI as an independent factor associated with lymph node metastasis; however, its applicability to microinvasive carcinoma is limited because only 64 patients had stage IA disease. More specifically, Margolis et al. [21] demonstrated an association between LVSI and lymph node involvement in stages IA1-IA2, supporting its use to guide nodal assessment rather than automatically escalating cervical surgery. Lécuru et al. [59], in the SENTICOL I study, demonstrated the high negative predictive value of bilateral mapping. Favre et al. [60] confirmed reduced morbidity without an evident compromise in oncological outcomes in the follow-up of SENTICOL II.

Ultrastaging, using serial sections and cytokeratin immunohistochemistry, increases the detection of micrometastases and isolated tumor cells [61,62,63]. If sentinel lymph node biopsy is performed, the ESGO/ESTRO/ESP guidelines recommend ultrastaging [6]. In microcarcinoma, however, the significance of isolated tumor cells and the effect on treatment remain uncertain; the procedure primarily improves diagnostic accuracy. Furthermore, SENTICOL I and II predominantly included early cervical tumors and were not sized for specific stage IA analyses: the application to microcarcinoma therefore remains partly an extrapolation. More recent prospective evidence from the SENTIX study further demonstrated the diagnostic value of pathological ultrastaging. Among 647 patients undergoing SLN ultrastaging, 81 (12.5%) had nodal involvement; importantly, ultrastaging identified additional metastatic disease in 35 of these 81 N1 cases (43.2%) that had not been detected by preoperative imaging and intraoperative pathological assessment. The detection rate increased with the intensity of pathological assessment, and examination of four levels identified more than 90% of N1 cases. These findings highlight the limitations of conventional staging in excluding occult low-volume nodal disease; however, because SENTIX included patients from T1a1/LVSI-positive to T1b2 disease, the applicability of these findings specifically to FIGO stage IA remains indirect [32].

The reliability of sentinel lymph node biopsy depends on adherence to the algorithm. Bilateral mapping is required; if no nodes are identified on one side of the pelvis, nodal evaluation should be completed on the unmapped side according to the center’s recommendations. Any macroscopically suspicious lymph node should be removed regardless of mapping. The advantage of this method is not only the reduction of lymphedema and neurovascular morbidity, but also the possibility of focusing the pathological examination on a few lymph nodes through ultrastaging [6,59,60,61,62,63,64,65,66,67,68].

The management of micrometastases and, especially, isolated tumor cells remains controversial. In microinvasive tumors, the low frequency of events makes it difficult to demonstrate their prognostic impact and determine whether they should modify treatment. The decision should not be automatic, but should consider the size of the deposit, LVSI, histological type, completeness of excision, and the potential consequences of further therapies.

Beyond nodal ultrastaging, emerging liquid-biopsy approaches are being investigated as potential tools for detecting tumor-derived material that may not be identifiable by conventional imaging or pathological assessment. Circulating tumor cells (CTCs), circulating tumor DNA (ctDNA), and circulating cell-free HPV DNA (cfHPV-DNA) have shown potential diagnostic and prognostic value in cervical cancer. Highly sensitive molecular techniques, including droplet digital PCR (ddPCR) and next-generation sequencing (NGS), have further improved their detection, with cfHPV-DNA emerging as one of the most promising circulating biomarkers for treatment monitoring and early detection of recurrence [69,70]. However, available studies involve heterogeneous cervical cancer populations, and these approaches have not been specifically validated for FIGO stage IA disease. Liquid biopsy should therefore currently be regarded as investigational and cannot replace pathological or nodal staging or be used to guide therapeutic de-escalation in microinvasive cervical cancer. This is one area in which prospective registries dedicated to stage IA could produce clinically relevant information (Table 3).

## 6. Follow-Up

Follow-up is an integral part of conservative treatment. It should verify oncological control, HPV persistence, cervical outcomes and reproductive or psychosocial needs. Guidelines recommend periodic history and pelvic examination, with cytology, HPV testing and colposcopy adjusted according to treatment and findings [6,7]. It is useful to integrate the use of biomarkers in cases of doubtful interpretation (Overexpression of E6-E7); however, their use in the follow-up of microinvasive carcinoma is not currently standardized [70]. Imaging is not routinely indicated in an asymptomatic patient with radically treated microcarcinoma; MRI, CT or PET/CT should be reserved for symptoms, suspicious findings or specific clinical risk [6,26,28,29].

After fertility-sparing, surveillance should be centralized and prolonged. Persistence of the same HPV genotype [71], a previously involved margin, LVSI, and difficulty visualizing the squamocolumnar junction warrant closer monitoring. Counseling should also address anxiety, pregnancy timing, and obstetric management, as successful treatment does not simply mean the absence of recurrence.

The frequency of checks should be adapted to the treatment and risk profile, avoiding rigid schedules not supported by specific evidence. In the first years, closer surveillance is reasonable, which can be reduced thereafter in the absence of findings. Cytology and HPV testing are complementary: a negative HPV result is reassuring, while persistence requires further colposcopic investigation. After hysterectomy, vaginal checks should be adjusted based on the history of HSIL/AIS and not confused with routine radiological surveillance [6,7,72,73,74,75].

A subsequent pregnancy does not eliminate the need for appropriate oncological evaluation, but it does require coordination. Before conception, it is advisable to document the absence of disease and cervical patency; during pregnancy, attention also shifts to cervical length and the risk of preterm birth. After delivery, the patient should re-enter the follow-up program, again discussing whether to maintain the conservative strategy or consider definitive treatment once the reproductive plan is complete.

## 7. Discussion

The literature converges on one principle: in microinvasive carcinoma, anatomical radicality can be replaced by diagnostic precision. Studies by Hartman et al. [14,34], Chen et al. [15] and Azzi et al. [33] show that, in IA1 without LVSI and with negative margins, conization offers a very high oncological control. The differences between the cold knife technique and the diathermic snare become secondary when the specimen is intact, the margins are evaluable and the invasion is measured correctly.

LVSI constitutes the main area of uncertainty. The data of Cao et al. [26] confirm its relationship with lymph node metastases, but derive mainly from higher stages; the analysis of Margolis et al. [27] is more pertinent to stage IA and supports the use of LVSI as an indicator for lymph node staging. An important distinction follows: positive LVSI may modify lymph node management without automatically imposing radical cervical surgery.

Even for the sentinel lymph node, the specific evidence is incomplete. SENTICOL I and II [59,60] claim accuracy and reduced morbidity in early carcinoma, but include few microinvasive cases. Therefore, the omission of staging in LVSI-negative IA1 is well established, while the indication for SLN in LVSI-positive IA1 and IA2 relies on guidelines and reasonable extrapolations, rather than on dedicated trials.

Fertility preservation has instead moved from an experimental option to an accepted strategy in selected patients. Zhang et al. [39], Wang et al. [40] and Gil-Ibañez et al. [41] document high survival, but the heterogeneity of the populations suggests not to extend the results beyond the limits of stage and histotype. The meta-analysis by Kyrgiou et al. [42] also recalls that the quality of survival includes obstetric outcomes: the minimum effective radicality should become an indicator of quality.

Finally, SHAPE and GOG-0278 [36,37] confirm the value of de-escalation even in women who do not wish to become pregnant. Although they include low-risk tumors that are not exclusively microinvasive, they strengthen the choice of simple hysterectomy and reduce the scope for radical surgery in correctly staged IA. The main limitations of this review are the narrative nature, the scarcity of studies exclusive to IA2, the predominance of retrospective data, and the heterogeneity of historical definitions.

Overall, a sequential care model emerges. Conization is not merely a therapeutic procedure, but the step that generates the information necessary for every subsequent decision. The pathology report identifies patients in whom treatment can be concluded and those who require re-excision, lymph node evaluation, or definitive surgery. Reproductive desire occurs after this stratification, not before: it modifies the treatment approach, but not the oncological safety requirements. The main contribution of this review is therefore not merely to summarize current management options for FIGO stage IA cervical cancer, but to critically define the boundaries of therapeutic de-escalation in microinvasive disease. By distinguishing evidence generated specifically in stage IA from that extrapolated from broader early-stage cervical cancer populations, we identify where conservative management is supported by robust evidence and where clinical decisions still rely on indirect data, expert consensus, or individualized risk assessment.

However, gray areas persist: the quantitative and qualitative significance of LVSI, specific indications for SLN in IA2, management of isolated tumor cells, the role of exclusive conization in IA2, and criteria for surgical completion after pregnancy. The rarity of the disease makes large, dedicated trials unlikely; prospective, multicenter studies, shared registries, and standardized reporting therefore represent the most realistic strategies to strengthen the evidence base. Patient-reported and obstetric outcomes should also be integrated with recurrence and survival.

## Figures and Tables

**Table 1:** Definition, diagnostic assessment, and key anatomopathological parameters in microinvasive cervical cancer.

Domain	Parameter	Clinical/Pathological Relevance
Definition	FIGO IA1	Stromal invasion ≤ 3 mm in depth
	FIGO IA2	Stromal invasion > 3 mm and ≤5 mm in depth
	Diagnosis	Histological diagnosis is required; stage IA cannot be assigned on clinical examination or imaging alone
Diagnostic excision	Conization	Provides definitive assessment of invasion, histotype, LVSI and margins; therefore, it has both diagnostic and potentially therapeutic value
	Specimen quality	An intact, well-oriented specimen with minimal thermal artifact is required for reliable measurement of invasion and margin assessment
Anatomopathological parameters	Depth of stromal invasion	Essential for distinction between IA1 and IA2
	Histological type	Squamous carcinoma vs. adenocarcinoma; HPV-associated vs. HPV-independent histotypes should be recognized
	LVSI	Does not modify FIGO stage but increases concern for lymph node involvement and influences nodal assessment
	Margins	Negative margins for invasive carcinoma and associated HSIL/AIS are required for conservative management
	Multifocality	Number and distribution of invasive foci should be reported and considered in pathological interpretation
	Tumor grade	Should be reported as part of complete pathological characterization
Additional risk assessment	Lymph-node status	SLN assessment may be considered according to stage and risk factors, particularly in IA2 and/or LVSI-positive disease
	Imaging	Limited role in correctly defined IA1; selectively used when more extensive disease or nodal involvement is suspected

**Table 2:** Risk-based therapeutic summary.

Scenario	Uterine Treatment	Lymph Node Evaluation
IA1, negative LVSI, negative margins	Conization if you want to have children; conization or simple hysterectomy if the reproductive plan is completed	Not indicated
IA1, LVSI positive	Conization or simple trachelectomy in selected cases; simple hysterectomy if not fertility-sparing	SLN to consider
IA2, favorable histology, negative margins	Wide conization/simple trachelectomy if fertility-sparing; simple hysterectomy in other cases	SLN recommended
Positive/non-assessable margins	Re-conization if feasible; subsequent treatment according to definitive histology	Second stage and LVSI
HPV-independent aggressive histology	Fertility-sparing not recommended	According to oncology guidelines

**Table 3:** Most informative clinical studies.

Study	Population/Design	Main Message
Hartman et al., 2017 [34]	FIGO IA1; long-term follow-up up to 20 years	Excellent long-term prognosis after appropriately selected conservative treatment
Hartman et al., 2021 [14]	562 patients with FIGO IA1; retrospective study	Cone dimensions and specimen quality influence margin status and reliability of pathological staging
Zhang et al., 2017 [39]	Fertility-sparing treatment; systematic review/meta-analysis	Low recurrence rates after fertility-sparing treatment, although evidence is limited by heterogeneity of observational studies
Lécuru et al., 2011—SENTICOL I [59]	Prospective multicenter study; early-stage cervical cancer	High diagnostic accuracy and negative predictive value of bilateral sentinel lymph node mapping
Kocian et al., 2024—SENTIX [32]	Prospective international study; 647 patients undergoing SLN ultrastaging	Ultrastaging identified additional nodal metastases in 43.2% of N1 cases not detected by preoperative imaging and intraoperative assessment; four ultrastaging levels detected >90% of N1 cases
Plante et al., 2024—SHAPE [36]	Randomized trial; low-risk early-stage cervical cancer ≤ 2 cm	Simple hysterectomy was non-inferior to radical hysterectomy, with lower surgical morbidity
Covens et al., 2025—GOG-0278 [37]	Prospective study; IA1 with LVSI to IB1	Supports feasibility of non-radical surgery and fertility-preserving approaches in carefully selected low-risk patients

## Data Availability

No new data were created or analyzed in this study. Data sharing is not applicable to this article.

## References

[1] World Health Organization (2026). Cervical Cancer.

[2] Sung H., Filho A.M., Laversanne M., Ferlay J., Siegel R.L., Soerjomataram I., Jemal A., Bray F. (2026). Global cancer statistics 2024: GLOBOCAN estimates of incidence and mortality worldwide for 34 cancers in 186 countries. CA A Cancer J. Clin..

[3] Ronco G., Dillner J., Elfström K.M., Tunesi S., Snijders P.J.F., Arbyn M., Kitchener H., Segnan N., Gilham C., Giorgi-Rossi P. (2014). Efficacy of HPV-based screening for prevention of invasive cervical cancer: Follow-up of four European randomised controlled trials. Lancet.

[4] Bhatla N., Berek J.S., Cuello Fredes M., Denny L.A., Grenman S., Karunaratne K., Kehoe S.T., Konishi I., Olawaiye A.B., Prat J. (2019). Revised FIGO staging for carcinoma of the cervix uteri. Int. J. Gynecol. Obstet..

[5] WHO Classification of Tumours Editorial Board (2020). Female Genital Tumours.

[6] Cibula D., Raspollini M.R., Planchamp F., Centeno C., Chargari C., Felix A., Fischerová D., Jahnn-Kuch D., Joly F., Kohler C. (2023). ESGO/ESTRO/ESP Guidelines for the management of patients with cervical cancer—Update 2023. Virchows Arch..

[7] National Comprehensive Cancer Network (2025). NCCN Clinical Practice Guidelines in Oncology: Cervical Cancer. Version 2025.

[8] Pecorelli S. (2009). Revised FIGO staging for carcinoma of the vulva, cervix, and endometrium. Int. J. Gynecol. Obstet..

[9] Bhatla N., Singhal S., Dhamija E., Mathur S., Natarajan J., Maheshwari A. (2021). Implications of the revised cervical cancer FIGO staging system. Indian J. Med. Res..

[10] Stolnicu S., Hoang L., Almadani N., De Brot L., Bovolim G., Baiocchi G., Brito M.J., Karpathiou G., Ieni A., Fernandez E.G. (2022). Horizontal tumor extent (HZTE) has limited prognostic significance in 2018 FIGO stage I endocervical adenocarcinoma (ECA): A retrospective study of 416 cases. J. Cancer Res. Clin. Oncol..

[11] Park K.J., Roma A., Singh N., Gilks C.B., Oliva E., Abu-Rustum N., Ramirez P.T., McCluggage W.G. (2021). Tumor Staging of Endocervical Adenocarcinoma: Recommendations From the International Society of Gynecological Pathologists. Int. J. Gynecol. Pathol..

[12] Bornstein J., Bentley J., Bösze P., Girardi F., Haefner H., Menton M., Perrotta M., Prendiville W., Russell P., Sideri M. (2012). 2011 colposcopic terminology of the International Federation for Cervical Pathology and Colposcopy. Obstet. Gynecol..

[13] McCluggage W.G., Judge M.J., Alvarado-Cabrero I., Duggan M.A., Horn L.-C., Hui P., Ordi J., Otis C.N., Park K.J., Plante M. (2018). Data set for the reporting of carcinomas of the cervix: Recommendations from the International Collaboration on Cancer Reporting (ICCR). Int. J. Gynecol. Pathol..

[14] Hartman C.A., Bragança J.F., Gurgel M.S.C., Zeferino L.C., Andrade L.A.L.A., Teixeira J.C. (2021). Conservative treatment of microinvasive squamous cell carcinoma of the cervix stage IA1: Defining conization height to an optimal oncological outcome. PLoS ONE.

[15] Chen S., Liu J., Shi C., Gao X., Bai H. (2021). The oncological and obstetric outcomes of cervical squamous cell carcinoma at stage IA1 managed with the loop electrosurgical excision procedure. Taiwan. J. Obstet. Gynecol..

[16] Yoneda J.Y., Bragança J.F., Sarian L.O., Borba P.P., Conceição J.C.J., Zeferino L.C. (2015). Surgical treatment of microinvasive cervical cancer: Analysis of pathologic features with implications on radicality. Int. J. Gynecol. Cancer.

[17] Lee S.J., Kim W.Y., Lee J.W., Kim H.S., Choi Y.L., Ahn G.H., Park N.H., Song Y.S. (2009). Conization using electrosurgical conization and cold coagulation for international federation of gynecology and obstetrics stage IA1 squamous cell carcinomas of the uterine cervix. Int. J. Gynecol. Cancer.

[18] Morris M., Mitchell M.F., Silva E.G., Copeland L.J., Gershenson D.M. (1993). Cervical conization as definitive therapy for early invasive squamous carcinoma of the cervix. Gynecol. Oncol..

[19] Lee K.B.M., Lee J.M., Park C.Y., Lee K.B., Cho H.Y., Ha S.Y. (2006). Lymph node metastasis and lymph vascular space invasion in microinvasive squamous cell carcinoma of the uterine cervix. Int. J. Gynecol. Cancer.

[20] Bisseling K.C.H.M., Bekkers R.L.M., Rome R.M., Quinn M.A. (2007). Treatment of microinvasive adenocarcinoma of the uterine cervix: A retrospective study and review of the literature. Gynecol. Oncol..

[21] Sopracordevole F., Clemente N., Alessandrini L., Di Giuseppe J., Ciavattini A. (2012). Conservative treatment of microinvasive adenocarcinoma of the uterine cervix: Long-term follow-up. J. Low. Genit. Tract Dis..

[22] Bruno M.T., Valenti G., Cassaro N., Palermo I., Incognito G.G., Cavallaro A.G., Sgalambro F., Panella M.M., Mereu L. (2024). The Coexistence of Cervical Intraepithelial Neoplasia (CIN3) and Adenocarcinoma In Situ (AIS) in LEEP Excisions Performed for CIN3. Cancers.

[23] Bumrungthai S., Ekalaksananan T., Evans M.F., Chopjitt P., Tangsiriwatthana T., Patarapadungkit N., Kleebkaow P., Luanratanakorn S., Kongyingyoes B., Worawichawong S. (2015). Up-Regulation of miR-21 Is Associated with Cervicitis and Human Papillomavirus Infection in Cervical Tissues. PLoS ONE.

[24] Xia P., Zhou J., Shen R., Wang D. (2025). Deciphering the cellular and molecular landscape of cervical cancer progression through single-cell and spatial transcriptomics. npj Precis. Oncol..

[25] Chen Q., Lyu J., Li Y., Zhang L., Zhao P., Gao G., Wang Y., Li C., Zhang X. (2026). Spatial transcriptomics of cervical cancer reveals microenvironment shaped the malignant epithelial programs and ECM-associated immune niches. Cancer Immunol. Immunother..

[26] Cao L., Kong W., Li J., Song D., Jin B., Liu T., Han C. (2023). Analysis of lymph node metastasis and risk factors in 975 patients with FIGO 2009 stage IA-IIA cervical cancer. Gynecol. Obstet. Investig..

[27] Margolis B., Cagle-Colon K., Chen L., Tergas A.I., Boyd L., Wright J.D. (2020). Prognostic significance of lymphovascular space invasion for stage IA1 and IA2 cervical cancer. Int. J. Gynecol. Cancer.

[28] Haldorsen I.S., Lura N., Blaakær J., Fischerova D., Werner H.M.J. (2019). What is the role of imaging at primary diagnostic work-up in uterine cervical cancer?. Curr. Oncol. Rep..

[29] Boemi S., Guagliardo G., Pasi S., Somma M., Pagana A., Bruno M.T. (2025). Magnetic Resonance Imaging in the Management of Women with Low-Risk Early-Stage Cervical Cancer: A Narrative Review. Diagnostics.

[30] Parra-Herran C., Malpica A., Oliva E., Zannoni G.F., Ramirez P.T., Rabban J.T. (2021). Endocervical Adenocarcinoma, Gross Examination, and Processing, Including Intraoperative Evaluation: Recommendations From the International Society of Gynecological Pathologists. Int. J. Gynecol. Pathol..

[31] Fischerová D., Cibula D., Stenhova H., Vondrichova H., Calda P., Zikan M., Freitag P., Slama J., Dundr P., Belacek J. (2008). Transrectal ultrasound and magnetic resonance imaging in staging of early cervical cancer. Int. J. Gynecol. Cancer.

[32] Kocian R., Kohler C., Bajsova S., Jarkovsky J., Zapardiel I., Di Martino G., van Lonkhuijzen L., Sehnal B., Sanchez O.A., Gil-Ibanez B. (2024). Sentinel lymph node pathological ultrastaging: Final outcome of the SENTIX prospective international study in patients with early-stage cervical cancer. Gynecol. Oncol..

[33] Azzi P.A., Russomano F.B., de Andrade C.V., de Camargo M.J., Jordão P.M. (2023). Frequency of post-treatment disease after excisional procedure in stage IA1 squamous cervical carcinoma: A case series. Women Health.

[34] Hartman C.A., Teixeira J.C., Barbosa S.B., Figueiredo S.M., Andrade L.A.L.D.A., Bastos J.F.B. (2017). Analysis of conservative surgical treatment and prognosis of microinvasive squamous cell carcinoma of the cervix stage IA1: Results of follow-up to 20 years. Int. J. Gynecol. Cancer.

[35] Bruno M.T., Valenti G., Ruggeri Z., Incognito G.G., Coretti P., Montana G.D., Panella M.M., Mereu L. (2024). Correlation of the HPV 16 Genotype Persistence in Women Undergoing LEEP for CIN3 with the Risk of CIN2+ Relapses in the First 18 Months of Follow-Up: A Multicenter Retrospective Study. Diagnostics.

[36] Plante M., Kwon J.S., Ferguson S., Samouëlian V., Ferron G., Maulard A., de Kroon C., Van Driel W., Tidy J., Williamson K. (2024). Simple versus radical hysterectomy in women with low-risk cervical cancer. N. Engl. J. Med..

[37] Covens A., Huang H.Q., Monk B.J., Kim Y.-B., Kim M.-H., DiSilvestro P., Vicus D., Holman L.L., Fleury A., Pearson J.M. (2025). Evaluation of efficacy and fertility after nonradical surgical therapy for stage IA1, IA2, and IB1 cervical cancer (GOG-0278). Gynecol. Oncol..

[38] Qian Q., Yang J., Cao D., You Y., Chen J., Shen K. (2014). Analysis of treatment modalities and prognosis on microinvasive cervical cancer: A 10-year cohort study in China. J. Gynecol. Oncol..

[39] Zhang Q., Li W., Kanis M.J., Qi G., Li M., Yang X., Kong B. (2017). Oncologic and obstetrical outcomes with fertility-sparing treatment of cervical cancer: A systematic review and meta-analysis. Oncotarget.

[40] Wang A., Cui G., Jin C., Wang Y., Tian X. (2019). Multicenter research on tumor and pregnancy outcomes in patients with early-stage cervical cancer after fertility-sparing surgery. J. Int. Med. Res..

[41] Gil-Ibañez B., Gil-Moreno A., Torné A., Martín Jimenez A., Gorostidi M., Zapardiel I., Tejerizo Garcia A., Diaz-Feijoo B. (2022). SEGO Spain-GOG Cervical Cancer Task Force. Tumor size and oncological outcomes in patients with early cervical cancer treated by fertility preservation surgery: A multicenter retrospective cohort study. Cancers.

[42] Nezhat C., Roman R.A., Rambhatla A., Nezhat F. (2020). Reproductive and oncologic outcomes after fertility-sparing surgery for early stage cervical cancer: A systematic review. Fertil. Steril..

[43] Kuznicki M.L., Chambers L.M., Morton M., Son J., Horowitz M., Crean-Tate K.K., Hackett L., Rose P.G. (2021). Fertility-sparing surgery for early-stage cervical cancer: A systematic review of the literature. J. Minim. Invasive Gynecol..

[44] Rob L., Skapa P., Robova H. (2011). Fertility-sparing surgery in patients with cervical cancer. Lancet Oncol..

[45] Bentivegna E., Maulard A., Pautier P., Chargari C., Gouy S., Morice P. (2016). Fertility results and pregnancy outcomes after conservative treatment of cervical cancer: A systematic review of the literature. Fertil. Steril..

[46] Plante M., Renaud M.C., Hoskins I.A., Roy M. (2005). Vaginal radical trachelectomy: A valuable fertility-preserving option in the management of early-stage cervical cancer. Gynecol. Oncol..

[47] Dargent D., Brun J.L., Roy M., Remy I. (1994). Pregnancies following radical trachelectomy for invasive cervical cancer. Gynecol. Oncol..

[48] Plante M., Renaud M.C., Sebastianelli A., Grégoire J. (2017). Simple Vaginal Trachelectomy: A Valuable Fertility-Preserving Option in Early-Stage Cervical Cancer. Int. J. Gynecol. Cancer.

[49] Schmeler K.M., Pareja R., Lopez Blanco A., Humberto Fregnani J., Lopes A., Perrotta M., Tsunoda A.T., Cantú-de-León D.F., Ramondetta L.M., Manchana T. (2021). ConCerv: A prospective trial of conservative surgery for low-risk early-stage cervical cancer. Int. J. Gynecol. Cancer.

[50] Schuurman T., Zilver S.J.M., Samuels S., Schats W., Amant F., van Trommel N.E., Lok C.A.R. (2021). Fertility-sparing surgery in gynecologic cancer: A systematic review. Cancers.

[51] Kyrgiou M., Athanasiou A., Paraskevaidi M., Mitra A., Kalliala I., Martin-Hirsch P., Arbyn M., Bennett P., Paraskevaidis E. (2016). Adverse obstetric outcomes after local treatment for cervical preinvasive and early invasive disease according to cone depth: Systematic review and meta-analysis. BMJ.

[52] Šimják P., Cibula D., Pařízek A., Sláma J. (2020). Management of pregnancy after fertility-sparing surgery for cervical cancer. Acta Obstet. ET Gynecol. Scand..

[53] Kwon J.S., McTaggart-Cowan H., Ferguson S.E., Samouëlian V., Lambaudie E., Guyon F., Tidy J., Williamson K., Gleeson N., de Kroon C. (2024). Cost-effectiveness analysis of simple hysterectomy compared to radical hysterectomy for early cervical cancer: Analysis from the GCIG/CCTG CX.5/SHAPE trial. J. Gynecol. Oncol..

[54] Bruno M.T., Ferrara M., Fava V., Barrasso G., Sapia F., Cutello S., Panella M. (2016). Prevalence Genotypes and Distribution of Human Papillomavirus Infection in Women with Abnormal Cervical Cytology in Catania, Italy. G. Ital. DI Ostet. E Ginecol..

[55] de Sanjose S., Quint W.G.V., Alemany L., Geraets D.T., Klaustermeier J.E., Lloveras B., Tous S., Felix A., Bravo L.E., Shin H.-R. (2010). Human papillomavirus genotype attribution in invasive cervical cancer: A retrospective cross-sectional worldwide study. Lancet Oncol..

[56] Bruno M.T., Panella M.M., Valenti G., Ruggeri Z., Sgalambro F., Reina S., Mereu L. (2024). Cervical Intraepithelial Neoplasia Grade 3 (CIN3) in Women Younger than 30 Years Was Significantly Associated with HPV16/18 Genotypes. Cancers.

[57] Monsonego J., Cox J.T., Behrens C., Sandri M., Franco E.L., Yap P.S., Huh W. (2015). Prevalence of high-risk human papilloma virus genotypes and associated risk of cervical precancerous lesions in a large U.S. screening population: Data from the ATHENA trial. Gynecol. Oncol..

[58] Reynolds E.A., Tierney K., Keeney G.L., Felix J.C., Weaver A.L., Roman L.D., Cliby W.A. (2010). Analysis of outcomes of microinvasive adenocarcinoma of the uterine cervix by treatment type. Obstet. Gynecol..

[59] Lécuru F., Mathevet P., Querleu D., Leblanc E., Morice P., Daraï E., Marret H., Magaud L., Gillaizeau F., Chatellier G. (2011). Bilateral negative sentinel nodes accurately predict absence of lymph node metastasis in early cervical cancer: Results of the SENTICOL study. J. Clin. Oncol..

[60] Favre G., Guani B., Balaya V., Magaud L., Lecuru F., Mathevet P. (2021). Sentinel lymph-node biopsy in early-stage cervical cancer: The 4-year follow-up results of the SENTICOL 2 trial. Front. Oncol..

[61] Balaya V., Guani B., Bonsang-Kitzis H., Deloménie M., Ngô C., Macias R.M., Koual M., Nguyen-Xuan H.-T., Bats A.S., Mathevet P. (2020). Sentinel lymph node biopsy in early-stage cervical cancer: Current state of art. Bull. Cancer.

[62] Bats A.S., Mathevet P., Buénerd A., Orliaguet I., Mery E., Zerdoud S., Le Frère-Belda M.A., Froissart M., Querleu D., Martinez A. (2013). The sentinel node technique detects unexpected drainage pathways and allows nodal ultrastaging in early cervical cancer: Insights from SENTICOL. Ann. Surg. Oncol..

[63] Guani B., Dorez M., Magaud L., Buenerd A., Lecuru F., Mathevet P. (2019). Impact of micrometastasis or isolated tumor cells on recurrence and survival in patients with early cervical cancer: SENTICOL trial. Cancers.

[64] Balaya V., Guani B., Magaud L., Bonsang-Kitzis H., Delomenie M., Montero Macias R., Ngô C., Mathevet P., Lécuru F. (2022). Long-term oncological safety of sentinel lymph node biopsy in early-stage cervical cancer: A prospective study. Eur. J. Cancer.

[65] Mathevet P., Guani B., Ciobanu A., Mery Lamarche E., Boutitie F., Balaya V., Lecuru F. (2021). Histopathologic Validation of the Sentinel Node Technique for Early-Stage Cervical Cancer Patients. Ann. Surg. Oncol..

[66] Cibula D., Kocian R., Plaikner A., Jarkovsky J., Klat J., Zapardiel I., Pilka R., Torne A., Sehnal B., Ostojich M. (2020). Sentinel lymph node mapping and intraoperative assessment in a prospective, international, multicentre observational trial of patients with cervical cancer: The SENTIX trial. Eur. J. Cancer.

[67] Balaya V., Guani B., Benoit L., Magaud L., Bonsang-Kitzis H., Ngô C., Le Frère-Belda M.A., Mathevet P., Lécuru F. (2020). Diagnostic value of frozen section examination of sentinel lymph nodes in early-stage cervical cancer at the time of ultrastaging. Gynecol. Oncol..

[68] Balaya V., Guani B., Magaud L., Bonsang-Kitzis H., Ngô C., Mathevet P., Lécuru F. (2020). Pre-operative surgical algorithm: Sentinel lymph node biopsy as predictor of parametrial involvement in early-stage cervical cancer. Gynecol. Oncol..

[69] Pineda-Migranas J.A., Bravata-Alcántara J.C., Cortés-Ortíz I.A., Cortés-Malagón E.M., Romero-Tlalolini M.d.L.Á., Sierra-Martínez M., Acosta-Altamirano G. (2025). Liquid Biopsy Biomarkers for Cervical Cancer: A Systematic Review. Int. J. Mol. Sci..

[70] Jiang J., Li L., Zhang C., Yang C., Dai Y., Chen Y., Huang Y., Xie L., Xiang Y., Yuan J. (2025). The application of liquid biopsy techniques in cervical cancer diagnosis, prediction and therapeutic surveillance. J. Gynecol. Oncol..

[71] Bruno M.T., Bonanno G., Sgalambro F., Cavallaro A., Boemi S. (2023). Overexpression of E6/E7 mRNA HPV Is a Prognostic Biomarker for Residual Disease Progression in Women Undergoing LEEP for Cervical Intraepithelial Neoplasia 3. Cancers.

[72] Bruno M.T., Valenti G., Cavallaro A.G., Palermo I., Aiello T., Farina J., Panella M.M., Mereu L. (2024). Extended Genotyping to Stratify the Risk of CIN2+ in Women with Persistent HPV Infection, Negative Cytology and Type Transformation Zone. Cancers.

[73] Elit L., Fyles A.W., Devries M.C., Oliver T.K., Fung-Kee-Fung M. (2009). Follow-up for women after treatment for cervical cancer: A systematic review. Gynecol. Oncol..

[74] Bruno M.T., Panella M.M., Valenti G., Di Grazia S., Sgalambro F., Farina J., Previti M., Mereu L. (2024). Vaginal Intraepithelial Neoplasia (VaIN) after Hysterectomy Is Strongly Associated with Persistent HR-HPV Infection. Cancers.

[75] Salani R., Khanna N., Frimer M., Bristow R.E., Chen L.M. (2017). An update on post-treatment surveillance and diagnosis of recurrence in women with gynecologic malignancies: Society of Gynecologic Oncology recommendations. Gynecol. Oncol..

